# Clinical usefulness of metagenomic next-generation sequencing for *Rickettsia* and *Coxiella burnetii* diagnosis

**DOI:** 10.1007/s10096-023-04586-w

**Published:** 2023-03-30

**Authors:** Xuan Zhang, Huixin Chen, Dongsheng Han, Wei Wu

**Affiliations:** 1 State Key Laboratory for Diagnosis and Treatment of Infectious Diseases, National Clinical Research Center for Infectious Diseases, National Medical Center for Infectious Diseases, Collaborative Innovation Center for Diagnosis and Treatment of Infectious Diseases, The First Affiliated Hospital, Zhejiang University School of Medicine, Hangzhou, 310003 China; 2 Department of Laboratory Medicine, the First Affiliated Hospital, Zhejiang University School of Medicine, Hangzhou, China; 3 Key Laboratory of Clinical In Vitro Diagnostic Techniques of Zhejiang Province, Hangzhou, China; 4 Institute of Laboratory Medicine, Zhejiang University, Hangzhou, China

**Keywords:** *Rickettsia*, *Coxiella burnetii*, Clinical characteristics, Metagenomic next-generation sequencing (mNGS), Diagnosis

## Abstract

*Rickettsia* and *Coxiella burnetii* are zoonotic tick-borne pathogens that cause febrile illnesses in humans. Metagenomic next-generation sequencing (mNGS) is a new technology used to diagnose infectious diseases. However, clinical experience with applying the test to rickettsioses and Q fever is relatively limited. Therefore, this study aimed to explore the diagnostic performance of mNGS in detecting *Rickettsia* and *C. burnetii*. We retrospectively studied patients with rickettsioses or Q fever between August 2021 and July 2022. Peripheral blood mNGS and polymerase chain reaction (PCR) were performed for all patients. Clinical data were retrieved for analysis. Thirteen patients were included in this study (eleven confirmed cases and two suspected cases). Signs and symptoms included fever (13, 100%), rash (7, 53.8%), muscle soreness (5, 38.5%), headache (4, 30.8%), skin eschar (3, 23.1%), and disturbance of consciousness (2, 15.4%). In addition, eight patients (61.6%) had thrombocytopenia, ten (76.9%) had liver function impairment, and two (15.4%) had renal function impairment. The results of mNGS revealed seven patients with *R. japonica* (53.8%), five with *C. burneti* (38.5%), two with *R. heilongjiangensis* (15.4%), and one with *R. honei* (7.7%). PCR results were positive in 11 patients (84.6%). After receiving doxycycline-based treatment, 12 (92.3%) patients returned to a normal temperature within 72 h. All patients were discharged in better health. Therefore, mNGS can help diagnose *Rickettsia and C. burnetii* and shorten the diagnosis time, especially for patients with atypical clinical manifestations and unclear epidemiologic evidence of a tick bite or exposure.

## Introduction

Rickettsioses are zoonoses transmitted by arthropods (including lice, fleas, ticks, and mites) worldwide. These zoonoses are among the oldest known vector-borne diseases [1]. The first clinical description of Rocky Mountain spotted fever, a prototypical tick-borne rickettsiosis, was published in 1899 [1]. In 1906, H.T. Ricketts reported the role of wood ticks in the transmission of the causative agent, subsequently named *Rickettsia rickettsia* [2]. Over the next 100 years, rickettsioses have been continuously studied worldwide. Q fever is a worldwide zoonosis caused by *Coxiella burnetii*, which has recently been removed from the order Rickettsiales.

Traditional methods for diagnosing rickettsioses include microbial culture, serological assays, and polymerase chain reaction (PCR)-based nucleic acid detection. However, these methods have some disadvantages. The culture of *Rickettsia* is restricted by time, a necessity for biosafety laboratory-3 facilities, and requires experienced personnel, which are not always appropriate for diagnosis [3]. Serological assays for the diagnosis of rickettsioses are the most frequently available and widely used worldwide [4] but do not provide species-level identification. The major concern for laboratories using PCR is the risk of contamination of lab/equipment with nucleic acids, leading to false positives [3]. Therefore, pathogen detection and identification methods for rickettsioses with high diagnostic efficiency are urgently required to overcome the current limitations of sensitivity, specificity, and timeliness.

The epidemiology of rickettsioses in China has been unclear over the past decade, and only a limited number of laboratory-confirmed cases have been reported. According to the database on the website of the Chinese Center for Disease Control and Prevention (CDC), the incidence of typhus in 2018 was 0.0699 per 100,000 people [5]. Most medical institutions do not provide appropriate testing in China; therefore, empirical treatment is conducted without a definitive diagnosis in clinical practice. In the absence of highly suggestive clinical manifestations(such as eschars) and clear epidemiological history, misdiagnoses and/or delayed diagnoses often occur, which may lead to severe, life-threatening complications for patients. Therefore, the need for appropriate detection and diagnostic strategies for identifying these organisms should not be underestimated.

Meta-genomic next-generation sequencing (mNGS) is a new technology that depicts promise in enhancing our capacity to diagnose infectious diseases [6–9]. Therefore, we conducted a retrospective study in China to analyze the diagnostic performance of mNGS in detecting *Rickettsia* and *C. burnetii* in adults.

## Materials and methods

### Study design and data collection

This retrospective observational study was conducted on patients with rickettsiosis or Q fever admitted to the First Affiliated Hospital, School of Medicine, Zhejiang University, from August 2021 to July 2022. Data were extracted from the electronic patient record system of the hospital. Demographic characteristics (age and sex), comorbidities, laboratory findings(routine blood test, high-sensitivity C-reactive protein [hsCRP], procalcitonin [PCT], liver function, renal function, and lactic dehydrogenase [LDH] levels), epidemiological history, clinical manifestations, treatment (antibiotic use and intensive care unit treatment), and prognosis were collected. Sequential Organ Failure Assessment (SOFA) was used as the prognostic scoring system. This study complied with the Declaration of Helsinki and was approved by the Ethics Committee of the First Affiliated Hospital of Zhejiang University. Because the study was retrospective and no identifiable patient information was included in this manuscript, the need for consent was waived.

## Enrollment criteria

The inclusion criteria for confirmed cases were as follows: (1) patients (≥ 18 years old) discharged from the hospital and diagnosed with rickettsiosis or Q fever at the First Affiliated Hospital, School of Medicine, Zhejiang University, from August 2021 to July 2022; (2) complete required data; (3) all patients underwent peripheral blood mNGS, which was performed by the laboratory department of the First Affiliated Hospital, School of Medicine, Zhejiang University; and (4) patients with positive PCR results and two infectious disease physicians confirmed the diagnosis based on historical epidemiology, clinical manifestations, and laboratory tests. The inclusion criteria for suspected cases were included the above criteria (1), (2), (3), and two infectious disease physicians confirmed the diagnosis based on historical epidemiology, clinical manifestations, laboratory tests, and therapeutic response.

## Real-time PCR

Real-time PCR was performed in all patients. The operation procedure was carried out according to the kit instructions (Zhijiang Bio, China). When the Ct value was less than or equal to 38, the result was recorded as positive. When the Ct value was 38–40, the retest was performed. If the Ct value was still greater than 38, the result was judged as negative. When the Ct value was undetected or 40, the result was negative.

## Metagenomic next‑generation sequencing and analysis

### Nucleic acid extraction, library preparation, and sequencing

DNA was extracted from peripheral blood using a QIAamp® UCP Pathogen DNA Kit (Qiagen), following the manufacturer’s instructions. Human DNA was removed using benzonase (Qiagen) and Tween20 (Sigma) [10]. Total RNA was extracted using a QIAamp® Viral RNA Kit (Qiagen), and ribosomal RNA was removed using a Ribo-Zero rRNA Removal Kit (Illumina). cDNA was generated using reverse transcriptase and dNTPs (Thermo Fisher). Libraries were constructed for DNA and cDNA samples using the Nextera XT DNA Library Prep Kit (Illumina, San Diego, CA) [11]. Library quality was assessed using the Qubit dsDNA HS Assay kit followed by a High Sensitivity DNA kit (Agilent) on an Agilent 2100 Bioanalyzer. Library pools were then loaded onto an Illumina Nextseq CN500 sequencer for 75 cycles of single-end sequencing to generate approximately 20 million reads for each library. For negative controls, we also prepared peripheral blood mononuclear cell samples with 105 cells/mL from healthy donors in parallel with each batch using the same protocol, and sterile deionized water was extracted alongside the specimens to serve as non-template controls (NTC) [11, 12].

### Bioinformatics analyses

Trimmomatic [13] was used to remove low-quality reads, adapter contamination, duplicate reads, and reads shorter than 50 bp. Low-complexity reads were removed by Kcomplexity using the default parameters [14]. Human sequence data were identified and excluded by mapping to a human reference genome (hg38) using Burrows-Wheeler Aligner software [15]. We designed a set of criteria similar to the National Center for Biotechnology Information (NCBI) criteria for selecting representative assemblies for microorganisms (bacteria, viruses, fungi, protozoa, and other multicellular eukaryotic pathogens) from the NCBI Nucleotide and Genome databases (https://www.ncbi.nlm.nih.gov/assembly/help/anomnotrefseq/) [16]. Pathogen lists were selected according to three references:1) Johns Hopkins ABX Guide (https://www.hopkinsguides.com/hopkins/index/Johns_Hopkins_ABX_Guide/Pathogens) [17], 2) Manual of Clinical Microbiology (https://www.clinmicronow.org/doi/book/10.1128/9781683670438.MCM)[18], and 3) clinical case reports or research articles published in peer-reviewed journals [19]. The final database consisted of approximately 13,000 genomes. Microbial reads were aligned to the database with SNAP v1.0beta.18 (https://arxiv.org/abs/1111.5572) [20]. Positive detection was reported for a given species or genus if the reads per million (RPM) ratio (RPM-r) was ≥ 5, where RPM-r was defined as the RPM sample/RPMNC (i.e., the RPM corresponding to a given species or genus in the clinical sample divided by the RPM in the negative control (NC)) [11]. In addition, to minimize cross-species misalignments among closely related microorganisms, we penalized (reduced) the RPM of microorganisms sharing a genus or family designation if the species or genus appeared in non-template controls. A penalty of 5% was used for species [21].

### Criteria for a positive mNGS result

For a given species that were also detected in the NC sample, we determined whether the species was detected by calculating a specifically mapped read number ratio (SMRN-r). SMRN-r was defined as the SMRN sample/SMRNNC (i.e., the SMRN corresponding to a given species in the clinical sample divided by the SMRN in the NC sample). If SMRN-r ≥ 10, the species was considered detected.

For an important human pathogen that is parasitic in cells, it is difficult to extract nucleic acids (e.g., *Rickettsia* app.) was considered positive when SMRN > 1. Therefore, *Rickettsia* and *C. burnetii* were considered positive when at least one read was mapped to either the species or genus level because of the difficulty of DNA extraction and low possibility of contamination.

## Results

### Clinical characteristics of the patients with rickettsiosis and Q fever

Thirteen patients with *Rickettsia* and *C. burnetii* detected by mNGS were included (Table 1). Of these, eleven patients were confirmed cases and two patients were suspected cases (no.2 and 9). The patients’ ages ranged between 35 and 78 years (mean, 59.2), with a male ratio of 84.6% (11/13). The vast majority of cases (12/13, 92.3%) occurred in spring and summer, mainly in April, May, and June (9/13, 69.2%). Two weeks before the onset of the disease, four patients (30.8%) were bitten by ticks, and nine (69.2%) had a history of farming, working in forestry, and walking in meadows and bushes.

**Table 1:** Clinical features of patients with *Rickettsia* and *Coxiella burnetii*

Case no	1	2	3	4	5	6	7	8	9	10	11	12	13
Age	50	46	68	67	70	78	51	35	65	58	66	70	45
Gender	male	male	male	female	male	male	male	male	male	male	male	male	male
Underlying diseases	hypertension	-	-	-	gout	Alzheimer's disease	hypertension	-	lung cancer	-	diabetes mellitus	multiple myeloma	-
Month of presentation	March	June	January	May	May	May	May	October	April	June	June	August	May
Tick bite	Yes	No	Yes	No	No	No	No	Yes	Yes	No	No	No	No
Exposure in the environment*	Yes	Yes	Yes	No	Yes	Yes	Yes	Yes	Yes	No	No	Yes	Yes
Symptoms and signs													
Fever	Yes	Yes	Yes	Yes	Yes	Yes	Yes	Yes	Yes	Yes	Yes	Yes	Yes
Rash	Yes	Yes	No	Yes	No	Yes	Yes	Yes	Yes	No	No	No	No
Muscle soreness	No	Yes	No	No	No	No	Yes	Yes	Yes	No	Yes	No	No
Headache	No	No	No	No	Yes	Yes	No	Yes	No	No	No	No	Yes
Skin eschar	No	Yes	No	No	No	Yes	No	No	No	Yes	No	No	No
Consciousness disorder	No	No	No	No	No	No	No	Yes	Yes	No	No	No	No
ICU treatment	No	No	Yes	No	No	No	No	Yes	Yes	No	No	No	No
Laboratory tests													
WBC (X10^9^/L)	12.65	8.65	10.99	13.17	10.03	16	8.57	5.64	10.6	3.56	2.39	5.16	4.43
Platelet (X10^9^/L)	52	218	1	65	22	33	89	96	258	71	87	56	110
hsCRP (mg/L)	91	116	17.5	43	129	73.9	213	114	50.7	81.3	46.3	173.6	63.6
PCT(ng/mL)	2.35	-	0.29	1.17	5.15	7.29	4.19	1.58	1.06	0.78	1.39	1.84	1.49
ALT(U/L)	64	53	14	40	29	28	500	42	45	74	31	89	102
AST(U/L)	76	52	20	39	84	93	687	65	48	127	21	90	68
Cr (µmol/L)	90	67	83	60	98	251	163	69	65	85	68	61	69
LDH(U/L)	464	336	538	449	536	640	908	348	374	673	301	251	357
SOFA	4	0	7	5	8	11	5	2	1	3	2	2	1
Hospital stay(days)	11	3	13	7	39	30	12	7	9	4	6	11	5

ICU, intensive care unit;WBC, White blood cells; hsCRP, high-sensitivity C-reactive protein; PCT, procalcitonin; ALT, alanine transaminase;AST, aspartate aminotransferase; Cr, creatinine;LDH, lactic dehydrogenase; SOFA, Sequential Organ Failure Assessment; *Farming, working in forestry, and walking in meadows and bush

All patients had a fever (13, 100%); rash, muscle soreness, headache, and skin eschar appeared in seven (53.8%), five (38.5%), four (30.8%), three (23.1%), respectively; two had altered consciousness (15.4%).

On admission, eight patients (61.6%) had thrombocytopenia; all patients (13, 100%) had significantly elevated hsCRP levels, 11 (84.6%) had elevated PCT; 10 (76.9%) had liver function impairment (aspartate aminotransferase > 40 U/L, alanine aminotransferase > 50 U/L), and two (15.4%) had renal function impairment (creatinine above the upper limit of normal).

**Fig. 1:**
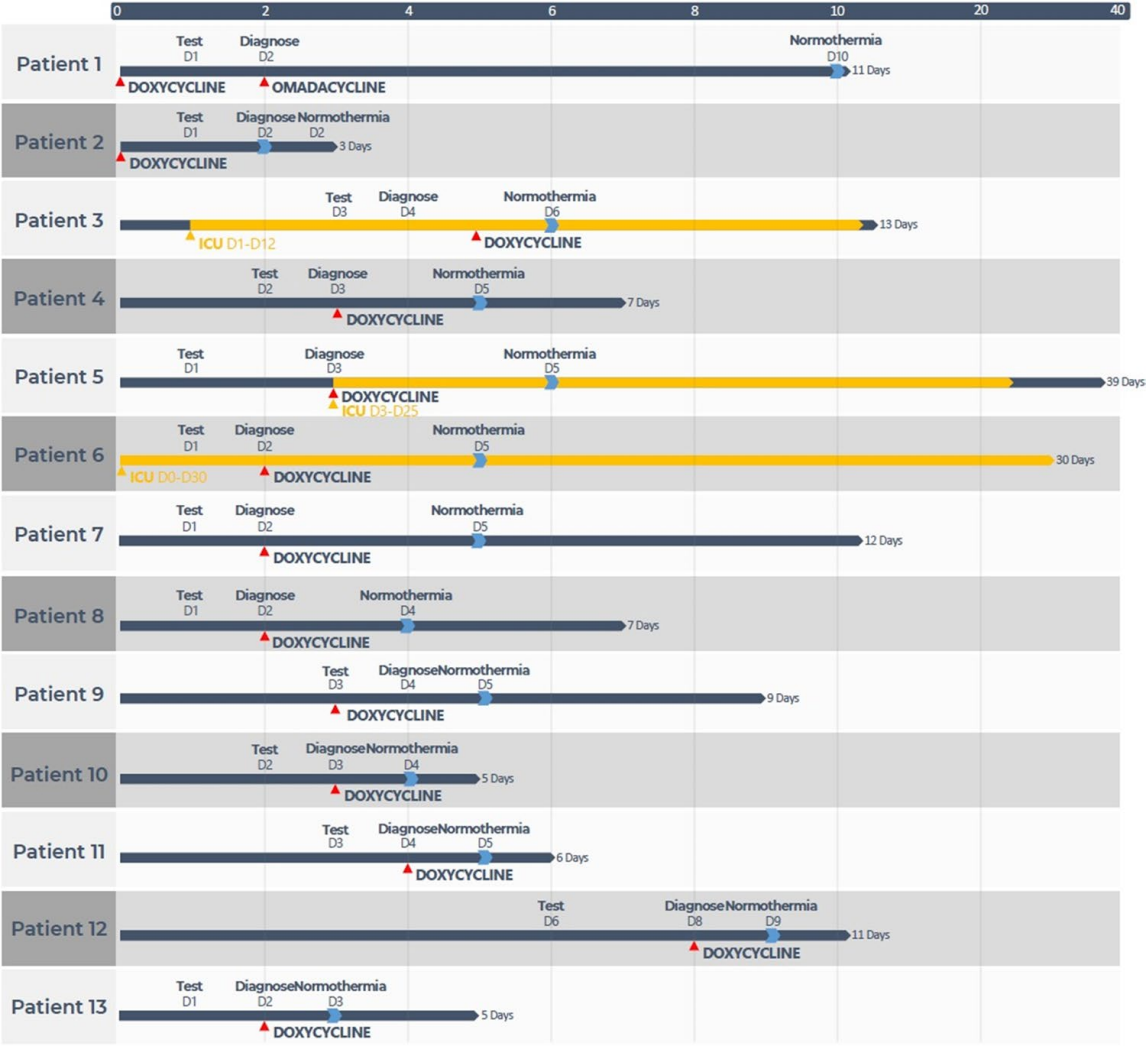
The timeline of the 13 patients with rickettsioses and Q fever during hospitalization. The timeline illustrates the different events during the patient’s treatment and disease progression. All patients were finally discharged in better health

The timeline of hospitalized patients is illustrated in Fig. 1. All patients received doxycycline-based anti-infective therapy (200 mg/day), and the temperature of 12 (92.3%) returned to normal within 72 h. Three patients (23.1%) were admitted to the intensive care unit for treatment, and all patients (100%) were finally discharged in better health.

## The results of mNGS and PCR

**Fig. 2:**
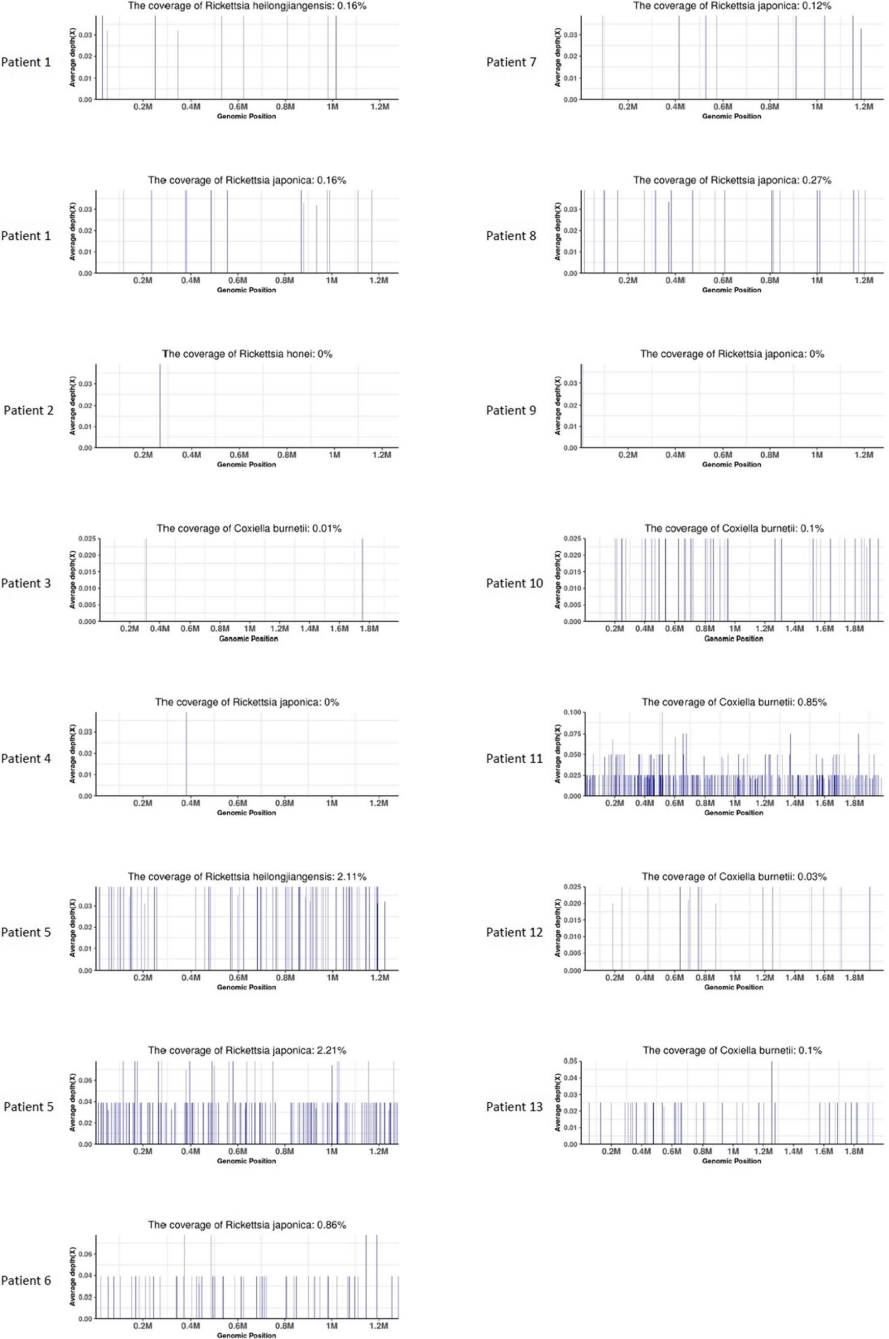
Mapping results of *Rickettsia* and *Coxiella burnetii* reads in whole blood

Among the 13 patients, mNGS results demonstrated seven with *R. japonica* (53.8%), five with *C. burnetii* (38.5%), two with *R. heilongjiangensis* (15.4%), and one with *R. honei* (7.7%). PCR results were positive in 11 patients (84.6%). Detailed results are displayed in Table 2. The mapping results of *Rickettsia* and *Coxiella burnetii* reads are illustrated in Fig. 2.

**Table 2:** The results of mNGS and PCR in patients with *Rickettsia* and *Coxiella burnetii*

Case no	1	2	3	4	5	6	7	8	9	10	11	12	13
*R. japonica*(reads)	10	-	-	1	100	36	10	9	1	-	-	-	-
*C. burnetii*(reads)	-	-	2	-	-	-	-	-	-	32	336	16	38
*R. heilongjiangensis*(reads)	7	-	-	-	43	-	-	-	-	-	-	-	-
*R. honei*(reads)	-	1	-	-	-	-	-	-	-	-	-	-	-
*human herpesvirus*(reads)	29	-	-	-	-	7	-	-	-	-	3	283	-
PCR(Ct value)	32.75	-	37.33	37.85	30.49	32.58	35.33	36.31	-	34.45	28.25	36.67	35.12

mNGS, metagenomic next-generation sequencing

In addition, mNGS results demonstrated the presence of human herpesvirus in whole blood samples from four patients (30.8%).

## Discussion

Our findings suggest that patients had acute infections with *Rickettsia* and *C. burnetii*. *Rickettsia japonica* and *C. burnetii* were the two most abundant pathogens in our study. *Rickettsia japonica* infection in humans has been reported in Anhui Province and Shandong Province of China [22, 23], which suggests that *R. japonica* is widely distributed in China. *Coxiella burnetii* is a pathogenic agent of Q fever. In China, the disease was initially reported in 1950, and there were 29 reports of Q fever in China between 1989 and 2013 [24]. However, five cases of Q fever were diagnosed in our study; perhaps the incidence of Q fever was grossly underestimated because of the difficulty of detection.

As described in previous studies, rickettsioses have no pathognomonic signs, although some signs and symptoms are highly suggestive, such as the presence of fever, rash, and eschar [25–27]. In the present study, fever was the most prominent manifestation. Skin eschar, as a relatively specific manifestation, occurs in less than 30% of cases. In addition, two weeks before disease onset, only a few patients reported tick bites. Therefore, it was difficult to diagnose rickettsiosis without performing laboratory tests. In the past decade, few large-scale epidemiological studies have been conducted on rickettsiosis in China. There are two possible reasons for this scarcity: First, there was no large-scale epidemic outbreak in China, and second, the detection means of medical institutions were limited. In 2010, the Chinese CDC successfully trained and popularized modern methods for diagnosing rickettsioses in ten provincial CDCs [28]. Unfortunately, in clinical practice, serological and PCR tests for *Rickettsia* remain unavailable in tertiary hospitals.

Metagenomic next-generation sequencing is a revolutionary technology that disrupts traditional clinical diagnostics on several fronts, such as microbiology [29]. The technology not only reduced the turnaround time but was also advantageous in testing clinical samples without any prior suspicion of certain pathogens required [30–32]. In our study, pathogens were detected by mNGS in all patients. *Rickettsia japonica* and *C. burnetii* were the two most abundant pathogens, but the mapping read numbers were relatively low. *Rickettsia* and *C. burnetii* are intracellular parasitic microorganisms. Compared with the common PCR detection method, in the detection process of plasma mNGS, the extracted nucleic acid was only free nucleic acid in plasma, which did not include the *Rickettsia* and *C. burnetii* genomes in blood cells. Therefore, the detectable nucleic acid load was low. Due to the difficulty of DNA extraction and the low possibility of contamination, *Rickettsia* and *C. burnetii*, similar to *mycobacterium tuberculosis* [9, 33], were considered positive when at least one read was mapped to either the species or genus level. Meanwhile, sequences of *Rickettsia* and *C. burnetii* were not detected in all of our negative samples (i.e., the "non-template controls" mentioned above) that were tested together with the clinical samples, which indicates that there are no false positives due to cross-contamination between samples. In this study, PCR results were positive in 11 patients, which was consistent with the results of mNGS. The remaining two patients were considered to have a rickettsial infection after the judgment of two infectious disease physicians based on epidemiological history, clinical symptoms, and effective treatment with doxycycline. Based on this, we considered that mNGS detection may be a complementary detection method for PCR detection, and it can rule out the possibility of other zoonotic tick-borne pathogens. As we know, the positive rate of pathogen detection could be improved when sampling before proper anti-infective treatment. Of the two PCR-negative patients, one patient was sampled after treatment, which may impact the detection of *Rickettsia*. Reports of the successful use of clinical metagenomics in the diagnosis of *Rickettsia* infection have been growing. A case reported that A middle-aged woman with fever, rash, and septic shock was diagnosed *R. honei* infection by sequencing methods on blood, while serologic tests for *Rickettsia* were negative [34]. Therefore, it is reasonable to assume that mNGS may be more sensitive in diagnosing *Rickettsia*. Although mNGS has advantages in diagnosing infectious diseases, it also has some disadvantages, such as high cost and the requirement of highly skilled personnel.

In our study, human herpes viremia was present in some patients, but it was not found to be the causative agent, which was also consistent with our previous knowledge that herpes virus presented as a latent infection.

Our study had some limitations. First, this was a retrospective, single-center study, and our study had a small sample size. Therefore, prospective studies are required to further investigate the diagnostic value of mNGS in *Rickettsia* and *C. burnetii*. Second, PCR was performed on patients in this study, but a few were negative, possibly because the blood samples were stored for different periods and the PCR kit had poor sensitivity. Finally, as the mNGS results may be easily influenced by many factors and there is no uniform standard for assaying positive results of rickettsioses in mNGS, the standards in our study should be tested in the future.

## Conclusion

Rickettsioses and Q fever are common zoonoses worldwide. Clinical manifestations such as fever and headache are nonspecific. If accompanied by tick bites or exposure to specific environments or animals, doxycycline can be recommended as an empirical treatment. Metagenomic next-generation sequencing can help diagnose rickettsioses and Q fever and shorten the diagnosis time, especially for patients with atypical clinical manifestations and unclear epidemiologic evidence of a tick bite or other exposure.

## Data Availability

The datasets generated during and/or analysed during the current study are available from the corresponding author on reasonable request.
